# Reframing chronic wound therapy: from growth factor delivery to regenerative immuno-engineering

**DOI:** 10.3389/fimmu.2025.1683591

**Published:** 2025-10-22

**Authors:** Zhiyong Wang, Xuan Zeng, Wenzhao Feng, Yanxin Lu, Pei Wei

**Affiliations:** ^1^ Department of Immunology, Zhuhai Campus of Zunyi Medical University, Zhuhai, China; ^2^ Department of Pharmaceutics, Guangdong Provincial People’s Hospital Zhuhai Hospital, Zhuhai, China; ^3^ College of Chemistry and Materials Science, Jinan University, Guangzhou, China

**Keywords:** chronic wounds, regenerative immuno-engineering, basic fibroblast growth factor, biomaterials, immunomodulation

## 1 Introduction

Chronic wounds represent a major global health crisis, fundamentally characterized by the failure of the immune system to resolve inflammation and transition to a pro-reparative state (1, 2). Chronic wounds impose a staggering global burden, subjecting hundreds of millions of patients to persistent pain, social isolation, elevated rates of depression, and a markedly increased risk of mortality, thereby severely compromising their quality of life (3, 4). In healthy acute wounds, the immune response comprises a precisely regulated sequence of events, initiating with an inflammatory phase for the clearance of debris and pathogens, and transitioning to an active resolution phase that orchestrates tissue regeneration (2, 5). In chronic wounds, this coordinated process becomes dysregulated, resulting in a state of persistent, low-grade inflammation. This immunological dysregulation is the central biological lesion (2, 6).

The clinical management of chronic wounds is traditionally guided by principles of standard care, often summarized by the TIME framework (Tissue debridement, Inflammation and infection control, Moisture balance, and Epidermal edge advancement) (7, 8). While essential for preparing the wound bed, these measures are often primarily supportive in nature (9). They manage the wound’s condition but frequently fail to actively trigger the stalled healing cascade in a biologically non-permissive environment (10). This insufficiency of standard care to overcome the intrinsic biological barriers of non-healing wounds provides the fundamental rationale for shifting toward active therapeutic approaches (10, 11). Consequently, the field has increasingly focused on strategies in bioengineering and tissue engineering, which aim to directly intervene in and modulate the biological processes of the wound to break the cycle of healing futility.

Within this paradigm of active therapeutic intervention, several major technological branches have emerged. These include (1): Cell-based therapies, which involve the application of allogeneic or autologous cells such as fibroblasts, keratinocytes, and stem cells, sometimes delivered within living cellular constructs (e.g., Apligraf^®^, Dermagraft^®^) (2, 12, 13); Tissue-engineered scaffolds, utilizing materials like acellular dermal matrices (ADMs) to provide a structural template for cellular infiltration and tissue regrowth (3, 14, 15) Bioactive molecule delivery, which uses biomaterial carriers to release signaling molecules like growth factors (15, 16). Among these diverse strategies, the approach centered on growth factor delivery has attracted an immense volume of research.

Yet paradoxically, despite its compelling reparative potential and a substantial research foundation, its clinical translation has been profoundly disappointing (17, 18). This translational gap reveals a critical paradox: within a persistent, pro-inflammatory microenvironment, the biological efficacy of exogenously administered pro-regenerative factors is severely compromised. This failure suggests that current research strategies may suffer from a fundamental limitation: they focus primarily on optimizing the technical parameters of drug delivery (a pharmaceutical engineering problem), while overlooking the fact that the core etiology of impaired chronic wound healing is immunological dysregulation. To further elucidate and validate this point, we use basic fibroblast growth factor (bFGF) as a model for three reasons. First, as a potent mitogen for fibroblasts and endothelial cells, bFGF promotes granulation tissue formation and angiogenesis, addressing two key obstacles in chronic wound repair. Second, bFGF is among the most intensively studied growth factors in sustained-release and nanocarrier research (19–22). Third, its well-defined molecular pharmacology—requiring heparin/heparan sulfate–mediated receptor dimerization and downstream signaling—makes it an ideal candidate for both advanced materials-based manipulation and for systematically dissecting how inflammation attenuates growth factor efficacy at the receptor, signaling, and extracellular matrix (ECM) levels.

**Figure 1:**
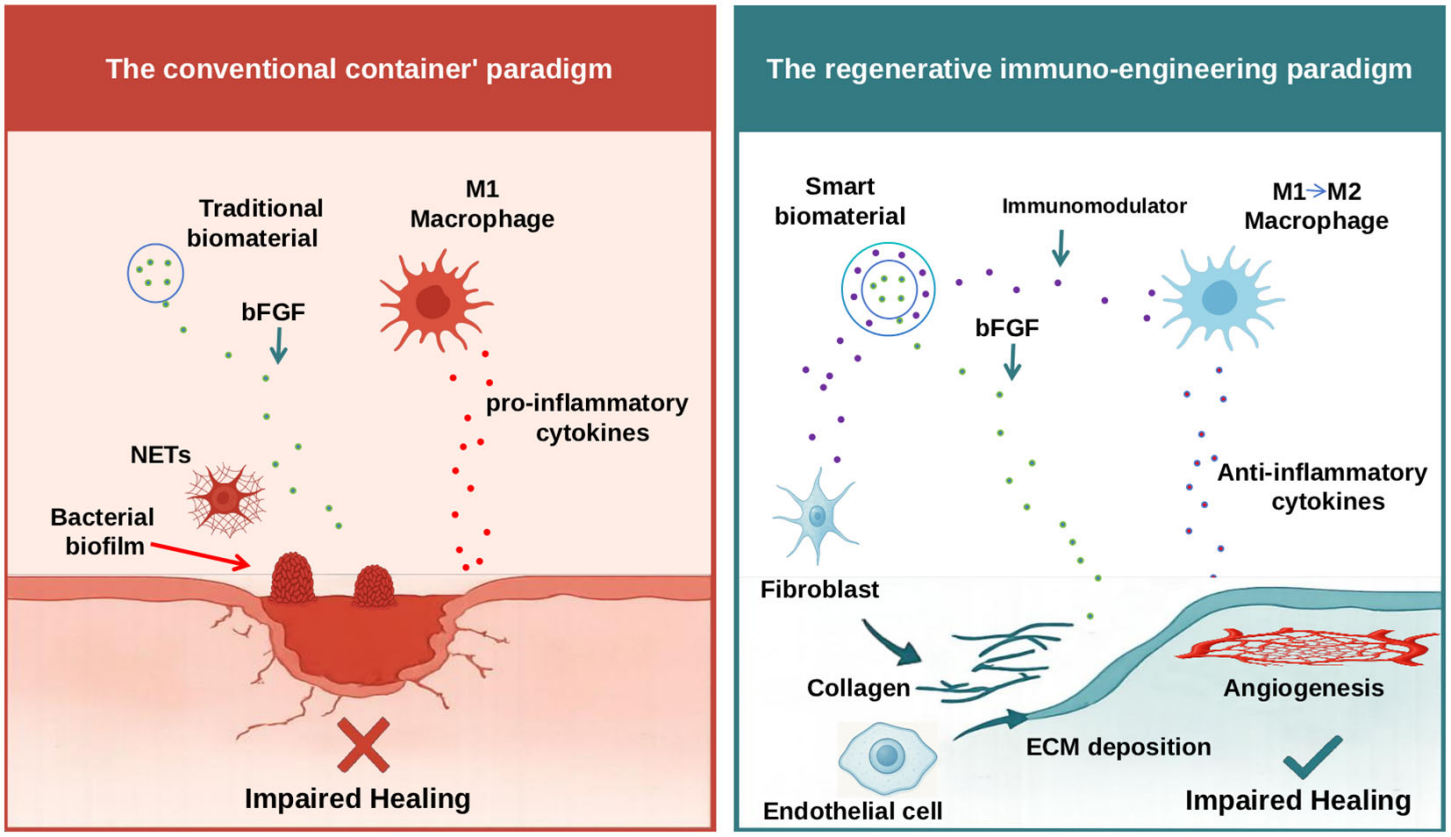
Schematic illustration of the conventional “container” paradigm versus the proposed regenerative immuno-engineering paradigm for chronic wound therapy. The left panel depicts the conventional “container” paradigm, where traditional biomaterials function as passive carriers, delivering bFGF into the chronic wound. However, this microenvironment is characterized by persistent, non-resolving inflammation, driven by pro-inflammatory M1-like macrophages secreting cytotoxic cytokines, excessive NETs, and the presence of bacterial biofilms. Despite bFGF delivery, this hostile inflammatory milieu largely renders the growth factor ineffective, leading to impaired healing. The right panel illustrates the regenerative immuno-engineering paradigm, a novel framework proposing the use of ‘smart biomaterials’ that first actively modulate the immune microenvironment. These smart biomaterials release immunomodulators to reprogram immune cells (e.g., promoting the phenotypic shift of M1 macrophages toward a pro-reparative M2 state, thereby facilitating the secretion of anti-inflammatory cytokines). Once immune homeostasis is restored and inflammation resolves, the biomaterial can then effectively deliver pro-regenerative factors like bFGF. In this permissive environment, bFGF can effectively stimulate fibroblasts to produce collagen and promote ECM deposition, and activate endothelial cells for angiogenesis, ultimately leading to enhanced tissue regeneration and successful wound healing.

In this opinion article, we contend that the persistent failure of growth factor-based therapies stems from a biologically naive “container paradigm” that prioritizes delivery engineering over fundamental immunology. We posit that the true obstacle to chronic wound healing is the underlying immune dysregulation, which renders pro-regenerative signals futile. Therefore, we advocate for a paradigm shift toward “regenerative immuno-engineering,” an approach that first actively modulates the immune microenvironment to resolve inflammation, thereby creating a permissive biological context for subsequent, precisely controlled regenerative cues (**Figure 1**). This article will critically dissect the biological pillars of failure in current approaches and then outline a new blueprint for regenerative immuno-engineering, emphasizing the strategic integration of immunomodulation with spatiotemporal control of healing signals. It should be emphasized that while bFGF serves as our central model, the immuno-engineering paradigm we propose is equally applicable to other regenerative strategies mentioned prior.

## 2 The “container” paradigm: a biologically agnostic approach

The prevailing research framework for bFGF delivery can be described as the “container” paradigm. The primary goal of this framework has been to engineer a physicochemically perfect vessel, optimizing for key metrics, such as loading efficiency, stability, and sustained-release kinetics from various platforms like hydrogels, nanofibers, or microspheres (23, 24). A representative study within this paradigm might report a high bFGF encapsulation efficiency of > 90%, accompanied by elegant graphs depicting a smooth, linear, zero-order release over 21 days *in vitro* (25, 26). These results, however, would be typically obtained under sterile, acellular, and protease-free phosphate-buffered saline conditions that bear no resemblance to the chaotic, hostile milieu of a real chronic wound (25, 26). This engineering-first approach is intellectually attractive but biologically naive. Furthermore, this approach operates on the flawed assumption that the chronic wound is a passive void, and that a constant supply of a pro-regenerative factor is sufficient to trigger healing.

This approach, while technically sound from a pharmaceutical engineering standpoint, commits a dangerous reductionist fallacy in that it attempts to address a dynamic, multifactorial, and systemic biological problem with a single, constant, and context-independent input (i.e., local bFGF concentration). This perspective systematically overlooks the complex and dynamic immune microenvironment of chronic wounds, which is characterized by the predominance of pro-inflammatory M1-like macrophages, excessive neutrophil extracellular traps (NETs) that cause collateral tissue damage, and a milieu of cytotoxic cytokines and proteases (6, 27, 28). In this specific context, any attempt to promote regeneration is futile. This carrier-centric research paradigm has failed because the pro-regenerative signals it delivers cannot be effectively received and transduced by target cells that exist in a pro-inflammatory state with altered signaling pathways.

## 3 The biological pillars of failure: an immunological re-examination

The clinical failure of growth factor-based therapies does not stem from an insufficient potency of molecules like bFGF itself, but rather from a profound immunological veto that systematically dismantles their regenerative potential at every critical step (2, 14). The “container paradigm” is inherently flawed because the pro-regenerative “message” it delivers is intercepted, corrupted, and ultimately ignored by target cells trapped in a non-resolving inflammatory state. This failure is built upon three interconnected pillars of immunological destruction.

First, the delivered growth factor is rapidly neutralized before it can reach its target. The microenvironment of chronic wounds is characterized by the infiltration and dominance of pro-inflammatory immune cells, particularly M1-like macrophages and hyperactivated neutrophils (28–31). These cells release a complex cocktail of destructive enzymes, such as high levels of matrix metalloproteinases (MMPs) (27, 32). Any exogenously delivered protein, including bFGF, is highly susceptible to degradation in such a proteolytically rich milieu. Furthermore, excessive Neutrophil Extracellular Traps (NETs)—web-like structures composed of DNA and cytotoxic proteins—not only cause collateral tissue damage but may also indirectly impede the effective diffusion and utilization of growth factors (28, 29). Consequently, in an environment that is both proteolytically active and presents dual physical and chemical barriers, even the most sophisticated sustained-release carriers cannot guarantee the integrity and bioavailability of their payload.

Second, even if exogenous bFGF escapes degradation through protective strategies, such as conjugation with heparin analogs (26), the signaling responsiveness of target cells is already significantly blunted by inflammation. The biological function of bFGF is highly dependent on its specific binding to its cognate receptor complex. This process is critically mediated by cell-surface heparan sulfate proteoglycans (HSPGs) (33, 34), which in turn triggers downstream intracellular signaling cascades, most notably the mitogen-activated protein kinase/extracellular signal–regulated kinase (MAPK/ERK) and phosphoinositide 3-kinase/protein kinase B (PI3K/AKT) pathways (35–37). Specifically, the MAPK/ERK pathway is a primary driver of cell proliferation and migration, stimulating the proliferation and motility of fibroblasts and keratinocytes, which are essential for granulation tissue formation and re-epithelialization (35, 36). Concurrently, the PI3K/AKT pathway plays a pivotal role in promoting cell survival by inhibiting apoptosis and regulating cell metabolism, while also fostering angiogenesis—all of which are indispensable processes for successful tissue regeneration (35, 37). The chronic wound microenvironment directly impairs or dismantles this signaling machinery. Activated immune cells, including M1 macrophages and neutrophils, secrete high levels of heparanase, and high expression of heparanase has been shown to degrade HSPGs in various models of chronic inflammation (38, 39). This creates a vicious cycle: inflammation destroys the very receptors required for a pro-regenerative response (40, 41). Concurrently, abundant pro-inflammatory cytokines in chronic wounds, such as tumor necrosis factor-α (TNF-α), can downregulate or interfere with several growth factor receptors and their downstream signaling, rendering cells refractory to proliferative/migratory stimuli (42, 43). Therefore, merely increasing the concentration of bFGF is a futile strategy when target cells have become insensitive or non-responsive to pro-regenerative stimuli due to persistent and strong inflammatory signal interference.

Finally, the entire cellular milieu is biologically misaligned and non-conducive to regeneration due to a profound failure in the process of “inflammation resolution.” Physiological healing is not merely the cessation of inflammation but a biochemically orchestrated program driven by specialized pro-resolving mediators (SPMs) that guides the cellular state from a pro-inflammatory phenotype to a pro-reparative phenotype (44–46). Chronic wounds are characterized by a severe deficit of these SPMs, leaving local cells trapped in a persistent, non-resolving state of inflammation (47–49). In this context, delivering a potent mitogen like bFGF via a passive carrier is biologically incoherent. It attempts to impose a proliferative signal upon an immune system and a stromal cell population that have not yet received the critical “stop inflammation” and “initiate resolution” commands. This mismatch does not lead to organized tissue regeneration but rather to aberrant, non-functional tissue deposition and potentially exacerbated fibrosis. In essence, the “container” approach fails because it attempts to initiate the tissue regeneration program before the critical biological prerequisite—the effective control and resolution of inflammation—has been met.

## 4 A new blueprint: principles of regenerative immuno-engineering

To break the translational stalemate, we must shift from the “container” paradigm to one of “Regenerative Immuno-engineering.” This approach views biomaterials not as carriers, but as active immunomodulatory platforms. The primary goal would be to re-establish immune homeostasis, thereby creating a permissive environment for endogenous and exogenous regenerative cues. This new framework is built on two core principles, as described below. **Table 1** summarizes representative engineering approaches that operationalize this regenerative immuno-engineering paradigm, organized by therapeutic principle, engineering modality, mechanism, and examples.

**Table 1:** Engineering approaches within the regenerative immuno-engineering paradigm.

Therapeutic principle	Engineering approach	Mechanism of action	Examples
Active immunomodulation	Biomaterial-mediated macrophage reprogramming	Delivery of signaling molecules to polarize macrophages from a pro-inflammatory M1 to a pro-reparative M2 phenotype.	Release of IL-4, IL-10, or M2 macrophage-derived exosomes.
	Functional materials targeting immune cells/factors	Inhibiting NETosis to reduce tissue damage; promoting Treg expansion for immune tolerance; sequestering excess pro-inflammatory cytokines.	NETosis-inhibiting materials; Treg-inducing signal delivery; “cytokine sponges” for TNF-α sequestration.
	Stimuli-responsive “smart” materials	Sensing pathological cues in the wound microenvironment and releasing therapeutics on-demand.	MMP-responsive release of anti-inflammatory drugs; ROS-responsive release of antioxidants.
	Antibacterial biomaterials	Eradicating bacteria and biofilms without inducing antibiotic resistance, thereby reducing the PAMPs load that drives M1 macrophage activation.	Photodynamic hydrogels; materials releasing NO or antimicrobial peptides.
Spatiotemporal control of signals	Sequential release systems	Mimicking the natural healing cascade by releasing different bioactive molecules in stages.	Multi-layered hydrogels with differential degradation rates; systems with staged release of IL-4 (early) and bFGF/VEGF (mid-phase).
	On-demand release systems	Enabling precise external control over the timing and dosage of therapeutic release via non-invasive stimuli.	Nanoparticles/hydrogels responsive to near-infrared light or ultrasound.
	Multi-compartment or layered platforms	Spatially segregating different therapeutic components to achieve complex spatiotemporal delivery profiles.	Microneedle arrays capable of layered and sequential drug release.

### 4.1 Active immunomodulation as a therapeutic prerequisite

The primary objective of any advanced wound dressing must be the active modulation of the immune microenvironment. Pioneering studies have already demonstrated the feasibility of this approach (50). Biomaterials have been successfully engineered to drive macrophage reprogramming, shifting them from the M1 to the M2 phenotype, a critical step in resolving inflammation (51–53). Beyond macrophage reprogramming, future bioregulators could target other key immune players, for instance by designing materials that inhibit NETosis, deliver signals to promote regulatory T cell (Treg) expansion, or act as “cytokine sponges” to sequester excess TNF-α from the wound bed. Other “smart” materials could sense the pathological hallmarks of a chronic wound, such as high MMP levels or reactive oxygen species (ROS), and respond by releasing anti-inflammatory drugs or antioxidants, respectively (54, 55). Materials with intrinsic antibacterial properties, such as photodynamic hydrogels, can combat biofilms without inducing antibiotic resistance (56–58). Crucially, these functionalities can be synergistic; for example, disrupting a biofilm not only removes a bacterial source but also reduces the pathogen-associated molecular patterns (PAMPs) load that drives M1 macrophage activation. These studies exemplify the new role of this biomaterial as an “intelligent bioregulator” that first pacifies the battlefield.

### 4.2 Spatiotemporal control of immuno-regenerative signals

Once the immune environment is normalized, regenerative signals could be deployed effectively. This would require a sophisticated, “two-step” therapeutic strategy. The first wave of signals should be immunomodulatory, aimed at promoting the resolution of inflammation. Only after this is achieved should the second wave, featuring pro-regenerative factors such as bFGF and vascular endothelial growth factor (VEGF), be released. This necessitates a move away from simple and sustained release toward dynamic and sequential delivery (59, 60). This strategy could be achieved through a variety of sophisticated engineering strategies, such as multi-layered hydrogels with differential degradation rates, smart nano-valves that respond to specific pH or enzymatic cues, or on-demand systems triggered by external stimuli such as light or ultrasound (61–63). An idealized release profile might involve three stages: an initial burst of immunomodulators (e.g., IL-4 or SPMs) for the first 0–3 days; the subsequent release of pro-angiogenic and proliferative factors (e.g., bFGF/VEGF) from day 3 to 10; and a final phase delivering anti-fibrotic or tissue-remodeling agents to ensure high-quality tissue formation. The engineering of multi-compartment or layered systems, such as advanced microneedle arrays, provides a tangible platform for achieving this critical level of spatiotemporal control (64, 65). This approach, validated by the quantitative monitoring of downstream signaling pathways such as p-ERK and p-AKT would ensure that the right signal is delivered at the right time, in concert with the evolving immunological state of the wound (66, 67).

## 5 Conclusion and future perspectives

The long-standing failure to translate bFGF nanodelivery systems into clinical reality is not an indictment of nanotechnology, but of a research paradigm that has been divorced from fundamental immunology. The future research direction requires a fundamental shift in perspective: from designing materials as passive carriers to engineering intelligent biomaterials capable of specific molecular interactions with the immune system.

### 5.1 Bridging the bench-to-bedside gap: the path forward

We now call for the establishment of a truly integrated field of “regenerative immuno-engineering,” in which materials scientists and immunologists will collaborate from day one. This new field must be supported by a revolution in preclinical modeling. We must acknowledge the profound limitations of traditional murine models, whose rapid and robust healing capacity often masks the pathologies that prevent healing in humans (68, 69). The path forward requires embracing more physiologically relevant platforms such as three-dimensional (3D)-bioprinted and immune-competent skin equivalents that incorporate senescent cells and biofilms, organ-on-a-chip systems for high-throughput screening and the real-time monitoring of immune-material interactions, and large animal models of diabetes that better recapitulate systemic metabolic dysregulation (70–72).

Furthermore, the path to clinical translation is paved with regulatory challenges. These drug-device combination products present unique hurdles for agencies. Key questions arise regarding the characterization of the material’s mechanism of action (is it a device, a drug, or both)?, the establishment of relevant biomarkers to prove *in vivo* immunomodulation, and ensuring lot-to-lot consistency for complex, multi-functional materials (73, 74). A “design-for-translation” approach that incorporates regulatory science early in the development process is essential.

### 5.2 Technological convergence for personalized wound management

When positioned against the broader landscape of advanced therapies, the rationale for regenerative immuno-engineering becomes even more compelling. Compared to cell-based therapies, which introduce exogenous living cells into a hostile environment, our approach offers a more fundamental solution. Instead of delivering cells that may struggle to survive and function amidst chronic inflammation, regenerative immuno-engineering first focuses on “detoxifying” the wound bed, creating a permissive niche where the patient’s own endogenous cells can be activated to drive repair. This “host-centric” strategy may also offer significant advantages in terms of cost, scalability, and off-the-shelf availability over complex cell logistics. Specifically, the “off-the-shelf” nature of these advanced biomaterials circumvents the complex logistics, high costs, and patient-specific manufacturing challenges associated with cell-based therapies. This inherent scalability makes the approach more amenable to widespread clinical adoption. Furthermore, while navigating the regulatory pathway for drug-device combination products is not without its hurdles, the potential for standardized, large-scale manufacturing and stringent quality control may present a more straightforward route to approval compared to the highly personalized and variable nature of many cell-based products. Similarly, while tissue-engineered scaffolds provide a valuable physical framework, they do not inherently address the underlying immunological paralysis. Regenerative immuno-engineering acts as a functional complement, transforming these passive scaffolds into active immunomodulatory platforms. In essence, rather than simply replacing or supplementing tissue components, our paradigm seeks to restore the intrinsic regenerative capacity of the host by correcting the root immunological defect.

Looking ahead, the “intelligent bioregulator” will serve as a platform for converging technologies. Artificial intelligence (AI) and machine learning, for instance, can move beyond simple screening to perform inverse design, predicting material compositions and surface topographies that will elicit a desired immune response (75, 76). We envision systems integrated with biosensors for closed-loop and adaptive therapy, and platforms that combine protein delivery with gene-based approaches to provide both short-term signals and long-term cellular programming (77, 78). Ultimately, this culminates in the vision of personalized wound management. For example, a patient’s wound exudate could be rapidly analyzed via single-cell or proteomic profiling to identify their unique “inflammatory signature,” which could then inform the AI-driven fabrication of a bespoke bioregulator dressing with a personalized cocktail of therapeutic agents and a tailored release schedule.

Confronting the manufacturing challenges will be paramount. However, the first and most crucial step is conceptual. We must recognize that healing a chronic wound is fundamentally an immunological challenge. Future biomaterials must not only deliver bFGF but also be capable of actively modulating the patient’s immune response through the release of specific signaling molecules. Only by imparting specific immunomodulatory functions to materials, enabling them to precisely intervene in the immune response process, can we expect to ultimately achieve coordinated and effective tissue regeneration.
